# Changing Trends in Liver Biopsy Practices: A Single-Center Analysis

**DOI:** 10.7759/cureus.46424

**Published:** 2023-10-03

**Authors:** Michael Makar, Umair Iqbal, Ayusa Sinha, Andrea Berger, Harshit S Khara, Bradley D Confer, Amitpal S Johal, Sandeep Khurana, David L Diehl

**Affiliations:** 1 Gastroenterology and Hepatology, Geisinger Medical Center, Danville, USA; 2 Internal Medicine, Geisinger Commonwealth School of Medicine, Danville, USA; 3 Biostatistics, Geisinger Medical Center, Danville, USA; 4 Gastroenterology and Nutrition, Geisinger Health System, Danville, USA

**Keywords:** transjugular liver biopsy, elevated liver enzymes, endoscopic ultrasound, percutaneous liver biopsy, liver biopsy

## Abstract

Introduction

To assess the trends for liver biopsy (LB) indications, technique, and histopathologic diagnosis, we retrospectively evaluated liver biopsies in two one-year periods, separated by a decade.

Methods

A pathology database query was performed for all parenchymal LB in patients over 18 years (11/2017 to 10/2018) and compared to those performed over a one-year period, a decade ago. We identified 427 parenchymal liver biopsies in the recent group and 166 in the decade-old group.

Results

Elevated liver enzymes are the most common indication for LB. Non-alcoholic fatty liver disease (NAFLD) has become the most common diagnosis compared to 10 years ago, when it was viral hepatitis. Routes of LB were significantly different between the two groups, endoscopic ultrasound-guided liver biopsy (EUS-LB) (80.3% vs 0; p<0.0001), computed tomography-guided (0 vs 42.8%, p<0.0001), percutaneous by gastroenterologists (0% vs 29.5%, p<0.0001), and transjugular-LB (15.1% vs 17.6%, p<0.0001). The adequacy of the tissue for pathological diagnosis was similar, and there was no difference in adverse events.

Conclusion

At our institution, practice patterns have changed significantly for liver biopsy. There has been an increase in liver biopsy volume, and EUS guidance has become the most common approach for liver biopsy.

## Introduction

Liver biopsy (LB) is the gold standard for the diagnosis of parenchymal liver diseases [1,2]. Common indications for liver biopsy include persistent elevation of liver enzymes and the diagnosis of various parenchymal liver diseases [3]. Historically, a percutaneous (PC) approach has been used [4,5]. A transjugular (TJ) route is preferred in patients with coagulopathy, significant ascites, or when measurement of the hepatic venous portal gradient is required [6,7]. Endoscopic ultrasound (EUS)-guided LB (EUS-LB) is an emerging technique with a diagnostic yield comparable to PC-LB and TJ-LB [8-10]. There are many potential advantages of EUS-LB, which is a sedated procedure that results in increased patient comfort and decreased procedure-related anxiety. In patients requiring a liver biopsy as well as an EGD or EUS, EUS-LB is more cost-effective and convenient as both are performed in a single session. EUS-LB also allows sampling of both lobes of the liver, decreasing sampling error. In addition, recovery time is shorter for EUS-LB (typically one hour).

At our institution, EUS-LB has expanded greatly over the last few years. To assess the evolving practice of liver biopsy, we retrospectively reviewed data on liver biopsies for two one-year periods separated by a decade, to determine the differences in indications, route of biopsy, adverse events, and final histopathologic diagnosis.

## Materials and methods

The study was conducted at a rural tertiary care hospital in the United States. A pathology database query was done for all LB in adult patients for the 12-month period from 11/2017 to 10/2018 (“2018”) and compared to the 12-month period from 11/2007 to 10/2008 (“2008”). Biopsies of focal hepatic masses were excluded. Data were collected regarding age, sex, body mass index (BMI), LB technique, adverse effects following LB, and referring specialty.

Data were analyzed using means and standard deviations for continuous variables and frequency counts and percentages for categorical variables. Characteristics of procedures performed in 2008 were compared to those in 2018 using Pearson’s chi-square or Fisher’s exact tests for categorical variables and student’s t-tests for continuous variables. Statistical analysis was completed using SAS software 9.4 (Cary, NC).

## Results

In 2008 and 2018, 166 and 427 LBs were performed, respectively. Baseline characteristics of these patients are shown in Table 1. Among the two groups, the patient’s mean age was similar. In 2018, the mean BMI was higher than that in 2008 (33.9 vs 30.5, p < 0.0001). In 2018, more women had liver biopsies (56.4% vs 46.4%, p = 0.0275). The route of biopsy, indication, and pathology results were significantly different among the two groups (all p < 0.0001). In 2008, 45.8% underwent LB for elevated liver enzymes, 33.1% for viral hepatitis, and 21.1% for other indications. In 2018, 68.6% had LB for elevated liver enzymes, 6.6% for viral hepatitis, and 24.8% for other indications. In 2008 the most common pathological diagnosis was viral hepatitis (39.76%) followed by NAFLD (38.5%). In 2018, the most common pathologic diagnosis was non-alcoholic fatty liver disease (NALFD) (55.7%) followed by viral hepatitis (14.2%). In 2008, 82.5% of patients were referred to gastroenterology or hepatology services, which increased to 93% in the more recent cohort.

**Table 1:** Summary of patient and procedure characteristics Bold = p < .05, which is clinically significant EUS-LB: endoscopic ultrasound-guided liver biopsy; DILI: drug-induced liver injury; NAFLD: non-alcoholic fatty liver disease

	2007-2008 (n = 166)		2017-2018 (n = 427)		P-value
	n	%	n	%	
Age, mean (S.D.)	51.2 (11.4)		53.0 (13.7)		0.1210
BMI, mean (S.D.) n missing = 12	30.5 (7.5)		33.9 (9.0)		< 0.0001
Ethnicity (n, %)					0.0528
African American	8	4.9%	7	1.6%	
Hispanic	2	1.2%	15	3.5%	
Caucasian	151	92.6%	402	94.1%	
Other	2	1.2%	3	0.7%	
Ethnicity (n, %)					0.4996
Non-Caucasian	12	7.4%	25	5.9%	
Caucasian	151	92.6%	402	94.1%	
Gender (n, %)					0.0275
Female	77	46.4%	241	56.4%	
Male	89	53.6%	186	43.6%	
Route (n, %)					< 0.0001
CT-Guided	71	42.8%	0	0.0%	
EUS-LB	0	0.0%	343	80.3%	
Fluoroscopy-Guided	5	3.0%	0	0.0%	
Percutaneous by GI	49	29.5%	0	0%	
Transjugular	25	15.1%	75	17.6%	
US-Guided by IR	16	9.6%	9	2.1%	
Indication (n, %)					< 0.0001
Elevated Liver Enzymes	76	45.8%	293	68.6%	
Viral Hepatitis	55	33.1%	28	6.6%	
Other	35	21.1%	106	24.8%	
Adverse Events (n, %)					1.0000
Yes	1	0.6%	2	0.5%	
Sample Adequacy (n, %)					0.07
Yes	163	98.2%	426	99.8%	
Pathology Results (n, %)					< 0.0001
DILI	5	3.0%	6	1.4%	
NAFLD	64	38.5%	238	55.7%	
Viral Hepatitis	66	39.76%	62	14.2%	
Normal	18	10.84%	24	5.62%	
Autoimmune Hepatitis	7	4.21%	4	0.93%	
Other	6	3.6%	93	21.77%	
Referral					< 0.0001
Gastroenterology/Hepatology (n, %)	137	82.5%	397	93.0%	
Internal Medicine (n, %)	0	0.0%	6	1.4%	
Other (n, %)	29	17.5%	24	5.6%	

**Figure 1:**
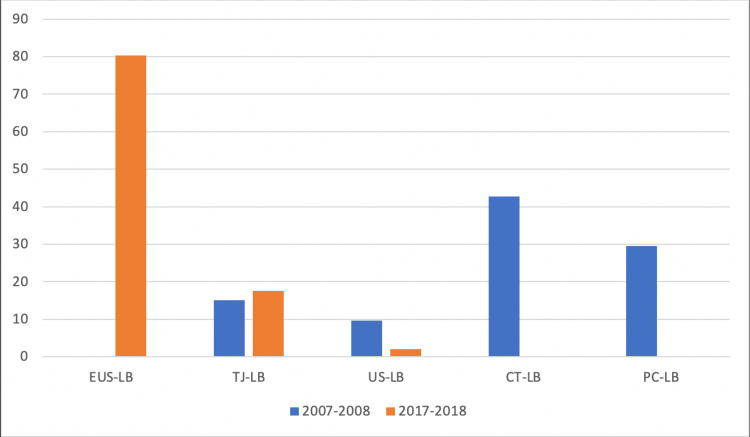
Preferred routes of liver biopsy during the two study periods EUS-LB: endoscopic ultrasound-guided liver biopsy; LB: liver biopsy; TJ: transjugular; PC: percutaneous

The route of biopsy was significantly different between the two time periods. In 2008, no patient had EUS-LB as compared to 80.3% in 2018. In patients who underwent EUS-LB, 45.7% also had concomitant indications for either EUS or EGD. The most frequent concomitant indications for EUS or EGD were dilated common bile duct/suspected choledocholithiasis, and assessment for evidence of portal hypertension. In 2008, 42.8% of patients had CT-guided LB as compared to none in 2018. In 2008, US-guided liver biopsy was performed by interventional radiology in 9.6% of patients compared to 2.1% of patients in 2018. In 2008, PC-LB was performed by gastroenterologists in 29.5% of patients compared to none in 2018 (Figure 1). Between the two groups, there were no differences in sample adequacy (98.2% in 2008 vs 99.8% in 2018; p=0.07). Three patients experienced adverse events. One patient had gastric mucosal bleeding after EUS-LB and was treated with three hemostatic clips. After TJ-LB, one patient developed atrial fibrillation with rapid ventricular rate. Another patient developed a distended abdomen secondary to subcapsular hematoma after CT-guided LB and required IR-guided embolization to control bleeding. There were no differences in adverse events between the two groups (0.6% in 2008 vs 0.5% in 2018; p=1.00). Table 1 summarizes differences in routes, indications, and pathological diagnosis of liver biopsies between the two groups.

## Discussion

Over the past 10 years at our center, referrals for liver biopsy have increased three-fold. The need for a liver biopsy for viral hepatitis decreased while indications for NAFLD-related liver disease increased markedly [10]. The rapid rise in the prevalence of NAFLD has driven much of the need for LB despite more use of non-invasive liver assessments such as transient elastography (FibroScan) [11-14]. However, newer data suggest that transient elastography can overestimate the severity of hepatic fibrosis [15], maintaining the need for biopsy as the most definitive test to assess liver fibrosis.

At our center, approaches such as PC-LB and CT-guided LB have significantly decreased and have been replaced by EUS-LB. In 2008, 70% of liver biopsies were performed by approaches used by the interventional radiology department. By 2018, 80% of LBs were performed in the GI endoscopy department. Previously published data has shown no difference in the adequacy of sampling or rate of adverse events in patients who underwent EUS-LB compared to other modalities [10,16]. A retrospective study of 175 patients revealed that liver samples obtained via EUS-LB are comparable to PC-LB and TJ-LB; if bi-lobar EUS-LB sampling was performed, the tissue yields of EUS-LB exceeded PC-LB and TJ-LB [10]. It should be noted that this study was performed before the advent of a 19-gauge (19G) core biopsy needle, which has been shown to be superior to a regular 19G fine-needle aspiration (FNA) needle for LB. A recent systematic review and meta-analysis of 437 patients revealed histologic diagnosis in 93.9% and adverse events in 2.3% of patients [8].

Compared to percutaneous biopsy, EUS-LB has the advantage of being able to obtain biopsies from both liver lobes, which can decrease the sampling error. Our group has shown that single-lobe liver biopsy may underestimate hepatic fibrosis in up to 21% of patients with nonalcoholic steatohepatitis (NASH) while bi-lobar EUS-LB improves the evaluation of disease activity and fibrosis [17]. Another advantage of EUS-LB is that it is performed under sedation, which maximizes patient comfort and satisfaction and minimizes anxiety [18]. For pediatric patients, it may be a preferred method of liver biopsy [19]. In patients requiring LB who also require another endoscopic procedure, EUS-LB is the most convenient and cost-effective way to achieve this.

The limitations of our study include the retrospective nature of the study. There may have been variability among ordering physicians that could affect the preferences for a certain procedure. An EUS liver biopsy may be limited to certain institutions and would not be an option in certain locations.

The emergence of EUS-LB has been a cornerstone of the recent field that has been termed “Endoscopic Hepatology” [20-23]. The recent emergence of techniques to assess portal and hepatic venous pressures by EUS has been the next important development in endoscopic hepatology [24,25]. In the first human study including 28 patients, Huang et al. reported EUS-guided portal pressure measurement with 100% success and no adverse events. The investigators used a 25G FNA needle and a novel manometer. A transgastric or transduodenal approach was utilized to access the portal vein and hepatic vein or inferior vena cava [24]. Recently, Zhang et al. reported EUS-guided portal pressure gradient measurement using a 22G FNA needle [25]. This study revealed a good correlation between the EUS-guided portal pressure gradient with the hepatic venous pressure gradient measured via a transjugular approach. Further EUS-based innovations, for example, EUS-based shear wave parenchymal assessment or portal vein sampling will continue the development of endoscopic hepatology.

## Conclusions

The need for liver biopsies continues to exist despite the decrease in viral hepatitis, mainly contributed to by the rising incidence of NAFLD. Due to coagulopathy or the need for portal pressure measurements, some liver biopsies will still require the TJ route but with the development of EUS-guided techniques to measure portal pressures, the role of TJ access is likely to continue to decline in the future. Therefore, we feel that our experience is indicative of larger trends in the expanding role of EUS in managing patients with chronic liver disease.
